# Characterization, Spatial Variation and Risk Assessment of Heavy Metals and a Metalloid in Surface Soils in Obuasi, Ghana

**DOI:** 10.5696/2156-9614-8.19.180902

**Published:** 2018-08-20

**Authors:** Osei Akoto, Nesta Bortey-Sam, Shouta M.M. Nakayama, Yoshinori Ikenaka, Elvis Baidoo, Joseph Apau, Jemima Tiwaa Marfo, Mayumi Ishizuka

**Affiliations:** 1 Department of Chemistry, Kwame Nkrumah University of Science and Technology, Kumasi, Ghana; 2 Laboratory of Toxicology, Graduate School of Veterinary Medicine, Hokkaido University, Kita 18, Nishi 9, Kita ku, Sapporo 060-0818, Japan

**Keywords:** metals, mining, hazard quotient, thematic maps, ecological, cancer risk

## Abstract

**Background.:**

Soil contamination with heavy metals and metalloids has become an increasingly important issue in recent years.

**Objectives.:**

The present study examines possible contamination of the environment with metals from gold mining activities in Obuasi, Ghana.

**Methods.:**

Soil samples were collected from commercial and residential areas and tailing dams in Obuasi in order to investigate the extent of lead (Pb), cadmium (Cd), mercury (Hg), copper (Cu), zinc (Zn), cobalt (Co), chromium (Cr), nickel (Ni) and arsenic (As) pollution, create thematic maps showing the extent of heavy metals pollution, identify the sources of pollution, and to assess risks to humans and the surrounding ecological system.

**Results.:**

Mean concentrations of metals from the study were found in the order of As > Cu > Zn > Cr > Ni > Pb > Co > Hg > Cd. The results showed that all communities were severely polluted with As, and distribution maps highlighted two hot spots at the sulfite treatment plant and Pompura treatment plant tailings dams. Additionally, the levels of Pb, Cu and Zn were elevated around the city center where vehicular traffic is very dense. Principal component analysis indicated that mining activities may have significantly contributed to metal levels in Obuasi soils. The potential ecological risk (RI) indicated that soils in 41% of the communities pose very high risks to the surrounding ecological system, 50% pose considerable risk, and 9% pose a moderate risk. Arsenic and Hg contributed 73 and 15% of the RI, respectively. The average hazard quotient due to soil As exposure was 2.51 ± 1.23 and ingestion of soils in 95% of the communities in the study area could pose non-carcinogenic health risks to children. Moreover, the average cancer risk for children from the communities was 1.13 × 10^−3^. Based on the United States Environmental Protection Agency (USEPA) recommendation for cancer risk of 10^−6^ to 10^−4^, the cancer risk for children (> 10^−3^) was higher in 45% of the studied communities.

**Conclusions.:**

The central part of the study area is polluted with Pb, Zn and Cu, and As pollution is severe in all of the studied communities. The RI from all study sites revealed very high risk to the ecological system, including mammals. There could be non-cancer and cancer risks to Obuasi residents due to ingestion of As-contaminated soils, and children are particularly vulnerable.

**Competing Interests.:**

The author declares no competing financial interests

## Introduction

Soil contamination with heavy metals and metalloids has become an increasingly important issue in recent years. High concentrations of toxic metals in soil can reduce soil fertility and lead to accumulations in food and drinking water, with the potential to endanger human health through ingestion and/or inhalation.^1–5^ Exposure to high concentrations of lead can affect the central nervous system and reduces intelligent quotient in humans.^4^ Intake of cadmium (Cd)-contaminated food causes acute gastrointestinal effects, and kidney damage has been reported with chronic exposures.^6^,^7^ Children are more susceptible to heavy metal poisoning because they have an active digestive system and higher rate of metal absorption.^4^ In addition to these toxicities, the non-biodegradable nature and long half-lives of these metals in soil also pose risks.^8–11^

Mining, smelting, vehicular emissions, industrial waste and other related activities are some of the major sources of heavy metals soils contamination.^8^,^12^,^13^ Numerous studies regarding metal contamination in mining and smelting areas have been performed in the Europe, the United States, and Africa.^12^,^14–19^

The mining sector in Ghana has seen extensive expansion over recent decades. This rapid development has resulted in the generation of toxic substances which pollute soil, rivers, streams and boreholes.^1^,^12^,^20^ Heavy metals and arsenic pollution in soils and other environmental media in Obuasi have been previously studied.^21–24^ Toxicological risks arise when soil arsenic (As) concentrations exceed 40 mg/kg.^25^ Studies have shown that prolonged exposure to As could lead to cancer, diabetes, thickening of the skin, nervous system disorders, liver disease and digestive system problems.^26^

There are limited published data on the risks of metal exposure in Obuasi municipality through soil exposure, and little action has been taken over the years to deal with this problem. The objectives of the present study were to investigate the extent of heavy metals and metalloid pollution in the topsoil of communities within the Obuasi municipality, to create thematic maps of metal concentrations in the study area using geographical information systems mapping techniques, to identify possible contamination sources and relationships between metals and soil properties, and to assess the risk of these metals to humans and the surrounding ecological system.

## Methods

The Obuasi municipality is situated at the southern end of the Ashanti Region at 06°12′00″N 01°41′00″W. It covers a land area of 162.4 km^2^, with 53 communities. Obuasi is the administrative capital where the lucrative Obuasi Gold Mine (Anglo Gold Ashanti) is also located. The municipality has an estimated resident population of 287,000.^27^ The topography is undulating with a semi-equatorial climate with a double rainfall regime. Mean monthly rainfall ranges from 3.5 (January) to 283 mm (July). The average annual temperature is 25.5°C with relative humidity between 75%–80% in the wet season.^28^ Obuasi has the richest gold deposits in West Africa, and mining (including artisanal and small-scale mining) began in the 17th century. Since the late 1890s, Obuasi has developed into a modern mining town and it is one of the most densely populated areas in Ghana. Due its mountainous landscape with limited flat land, many of the residential and commercial structures are constructed on hillsides and on the flat areas within mountainous areas. Communities are therefore susceptible to pollution from various sources, including mining.

**Abbreviations**
*AAS*: Atomic absorption spectrophotometry
*dw*: Dry weight
*HQ*: Hazard quotient
*RI*: Ecological risk

## Sampling, sample preparation and analysis

**Figure 1:**
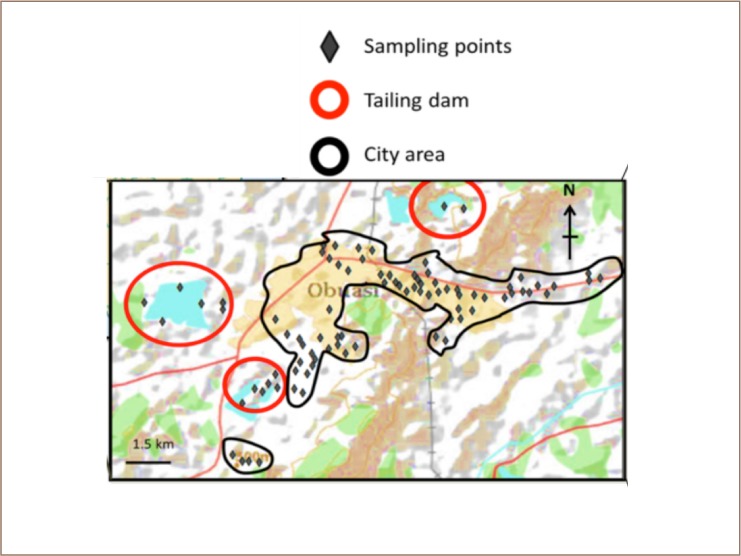
Map of study area showing the sampling points

As shown in Figure 1, twenty communities and two tailing dams were selected for the present study. The sampling sites/points were selected to represent a wide area of the town (*Figure 1*) and geographical coordinates for each site were recorded with a Garmin (etrex) global position system. A total of 86 surface soil samples were collected from open spaces within commercial and residential areas and tailings dams. The top soil layer was collected to a depth of about 10 cm with a stainless-steel spatula, as anthropogenic sources of pollutants usually contaminate the upper layer of soil.^29^

The collected samples were placed in labeled Corning tubes (Corning Incorporated, New York, USA) and transported to the toxicology laboratory at the Graduate School of Veterinary Medicine, Hokkaido University, Japan where they were stored at −30°C until analysis. Due to lack of background concentrations in soils in Obuasi, Ghana, data from 5 soil samples from the University of Mines and Technology campus in Tarkwa, Ghana, (Tarkwa is another mining community in Ghana) were used as reference values to evaluate the extent of metal pollution in the present study.^1^ University of Mines and Technology is a public university located in Tarkwa, and because it has low vehicular and industrial (mining) activities, heavy metals and metalloids from point sources were assumed to be negligible.^1^ Additionally, concentrations of metals in University of Mines and Technology were all low compared to the world range for unpolluted soils described by Kabata-Pendias and Pendias.^30^

Prior to chemical analysis, soil samples were air dried. Roots, stones and other unwanted materials were removed and remaining soil samples crushed. The homogenized samples were sieved through a 2-mm mesh. Soil samples were digested using a microwave digestion system (Speedwave MWS-2, Berghof, Germany) equipped with temperature and pressure feedback controls. Metals were digested by weighing 1 g of dried soil into pre-washed digestion vessels. Then 10.0 mL of 60% nitric acid (HNO_3_) (atomic absorption spectrometry grade; Kanto Chemical Corporation, Tokyo, Japan) was added. The vessels and content were covered and placed in the microwave oven for digestion. The microwave unit was calibrated to a temperature of 200^°^C and digestion was allowed for 45 minutes at 180 psi. After digestion, the solutions were allowed to cool and filtered using ash-less filter paper 5B (Advantec, Tokyo, Japan) into Corning tubes. Lanthanum chloride (1 mL, atomic absorption spectrometry grade, 100 g La/L solution, Wako Pure Chemical Industries Ltd., Osaka, Japan) was added to prevent physical, chemical and ionization/interference during metal analysis with atomic absorption spectrophotometry (AAS). Samples were diluted to a volume of 50 mL with 2% HNO_3_ prepared with milli-Q water. Reagent blanks were prepared using the same procedure. Concentrations of chromium (Cr), copper (Cu), As, Cd, cobalt (Co), nickel (Ni), lead (Pb) and zinc (Zn) were determined by AAS (Z-2010, Hitachi High Technologies Corporation, Tokyo, Japan) with either the acetylene flame or argon non-flame method, after preparation of calibration standards. Cadmium, Cr, Ni, Pb and As were analyzed by graphite furnace AAS with Zeeman background correction, while Cu and Zn were analyzed by flame AAS with deuterium background correction. The concentration of total mercury (Hg) was measured by thermal decomposition, gold amalgamation and AAS (Mercury Analyzer, MA–3000, Nippon Instruments Corporation, Tokyo, Japan), after preparation of calibration standards.

The water content of each soil sample was measured after 12 hours of drying in an oven at 105°C. Soil organic matter (soil organic matter) content was determined by loss of weight on ignition at an oven temperature of 600°C for 5 hours. pH was measured in a soil deionized water suspension (soil: water, 1:2.5 by volume) by a calibrated pH meter.

## Quality control and quality assurance

For quality control, blanks were analyzed after analysis of every 10 samples. Soil recovery rates (%) using certified reference materials (BCR-320R and SRM 1944) were: Cr (80–115), Co (96–111), Cu (88–95), Zn (90–95), Cd (113–120), Pb (91–114), Ni (85–87) and As (113–121). The detection limits (mg/kg) were 0.5 for Cr, 0.5 for Co, 1.0 for Cu, 0.1 for Zn, 0.2 for Cd, 1.0 for Pb, 0.5 for Ni and 2.0 for As. The detection limit of Hg in soil samples was 2.0 pg total Hg. Concentrations of metals and the studied metalloid in soils were expressed in mg/kg dry weight (dw).

## Data analysis

Potential ecological risk (RI) is a commonly used indicator to assess the effects of heavy metals and metalloids in the environment, including soils and sediments. The RI was calculated using Equations 1 through 4:

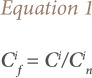


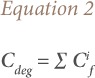


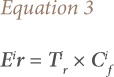


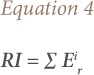
Where C^i^_f_ is the pollution coefficient of a metal which can reflect the pollution character of the investigated region but cannot reveal the ecological effects.^31^,^32^ C^i^ is the concentration of metals in soils. C^i^_n_ is the reference values of the heavy metals in soil/sediments. The C^i^_f_ of each metal was calculated and classified as either low (C^i^_f_ ≤ 1), middle (1 < C^i^_f_ ≤ 3) or high (C^i^_f_ > 3).^33^

C_deg_ represents the integrated pollution level in the environment and is expressed as the sum of C^i^_f_ for all examined metals. The four pollution levels were categorized as C_deg_ < 5, low pollution; 5 ≤ C_deg_ < 10, medium pollution; 10 ≤ C_deg_ < 20, high pollution; and C_deg_ ≥ 20, very high pollution.^34^ E^i^_r_ is the monomial potential ecological risk factor of the individual metal and T^i^_r_ is the metal toxic factor (based on the standardized heavy metal toxic factor). Referring to Hakanson, we used the following T^i^_r_ values: Hg = 40; Cd = 30; As = 10; Cu = Pb = Ni = 5, Cr = 2, and Zn = 1.^31^ The RI is defined as the sum of E^i^_r_ for all metals and has been grouped into four categories by Hakanson as shown in Table 1.^31^

**Table 1:** Single Factor and General Potential Ecological Risk Levels

*E^i^_r_*	Single factor pollution ecological risk level	RI value	General potential ecological risk level
*E^i^_r_* < 40	Low risk	RI ≤ 150	Low risk
*40 ≤ E^i^_r_* < 80	Moderate risk	150 < RI ≤ 300	Moderate risk
*80 ≤ E^i^_r_* < 160	Considerable risk	300 < RI ≤ 600	Considerable risk
*E^i^_r_* < 320	High risk	RI < 600	Very high risk
*E^i^_r_* ≥ 320	Very high risk		

*Abbreviations: E^i^_r_*, monomial potential ecological risk factor; RI, potential ecological risk.

Source: Hakanson, 1980^31^

### Human health risk assessment

Soil ingestion is one of the most common pathways through which humans, especially children, are exposed to metals.^35^ The risk due to metals was therefore assessed via oral ingestion. For incidental soil ingestion, Equation 5 were used according to the United States Environmental Protection Agency (USEPA).^36^

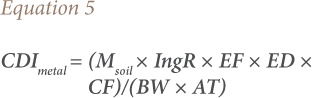
Where CDI_metal_ = metal daily intake (mg/kg/day); Msoil = average metal concentration in soil (mg/kg); IngR = ingestion rate of soil (mg/day); EF = exposure frequency (day/year); ED = exposure duration (year); BW = body weight (kg); AT = averaging time (days); and CF = conversion factor (10^−6^ kg/mg).^36^ The above equation assumes 100% bioavailability of the ingested soil-borne contaminant. Carcinogenic risk was determined by Equation 6.

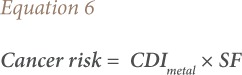



SF is the cancer slope factor (mg/kg/day)^−1^.^36^ For non-cancer risk, the hazard quotient (HQ) was calculated according to Equation 7 below, where RfD is the reference dose (mg/kg/day).^36^ All parameters used in the present study have been previously described.^37^

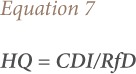



For the risk assessment, an HQ value greater than 1 indicates the potential for non-carcinogenic health effects, while HQ < 1 means residents are not likely to experience any health risks as a result of exposure.^38^ The acceptable or tolerable cancer risk for regulatory purposes is within the range of 10^−6^–10^−4^.^38^

### Statistical analysis

Statistical analyses were performed using statistical analysis software (SPSS) 20.0 (IBM SPSS Inc., Chicago, USA). Kolmogorov–Smirnov (K-S) and Shapiro–Wilk's tests were used to determine the normality of data and results were considered to be statistically significant if the p value was less than 0.05. Statistical analyses were carried out after data were log transformed (normalized). Spatial distributions were performed using ArcGIS 9.3 (ESRI Co., Redlands, USA). In order to identify the important parameters affecting the chemistry of soil and to investigate the possible sources of these metals, Pearson's correlation matrix (at a significance level of p<0.05) and principal component analysis were used, respectively. The principal components based on log transformed data were extracted with eigenvalues >1 through a varimax rotation.

## Results

Average concentrations of heavy metals and a metalloid (As) in soils in communities within the Obuasi municipality are shown in Supplemental Material 1. Mean concentrations (mg/kg dw) of metals decreased in the order As > Cu > Zn > Cr > Ni > Pb > Co > Hg > Cd (*Supplemental Material 1*). Kolmogorov–Smirnov and Shipro-Wilks statistical tests showed a significant distribution (p 0.05) of metals in the communities (*Supplemental Material 1*). Arsenic, Pb and Hg were classified as the 1st, 2nd and 3rd most hazardous substances.^39^ Cadmium (7th) was excluded due to low concentrations throughout the study area.

An overall examination of As distribution in soil found that the highest mean concentration (598 ± 132 mg/kg dw) was detected in samples from the Pompura Treatment Plant tailings dam, while the lowest (89.7 ± 58.2 mg/kg dw) was detected in samples from Sanso (*Supplemental Material 1*). Levels of As throughout the entire study area were extremely high compared to the world range for unpolluted soils and USEPA recommended levels (*Supplemental Material 1*), and could pose toxicological risks in the study area.^25^,^30^,^40^,^41^

As shown in Supplemental Material 1, mean Pb content in soil ranged from 4.30 ± 0.719 (Danquah Estate) to 189 ± 131 (Bedieso) mg/kg dw. The highest mean concentration of Hg was recorded at Sanso (5.19 ± 7.28 mg/kg dw) and lowest was 0.05 ± 0.01 mg/kg dw in Danquah Estate. Mercury pollution could well be underestimated in this study as small-scale gold miners using amalgamation is widespread in the area and no samples were collected from small-scale mining facilities.

### Distribution maps

**Figure 2:**
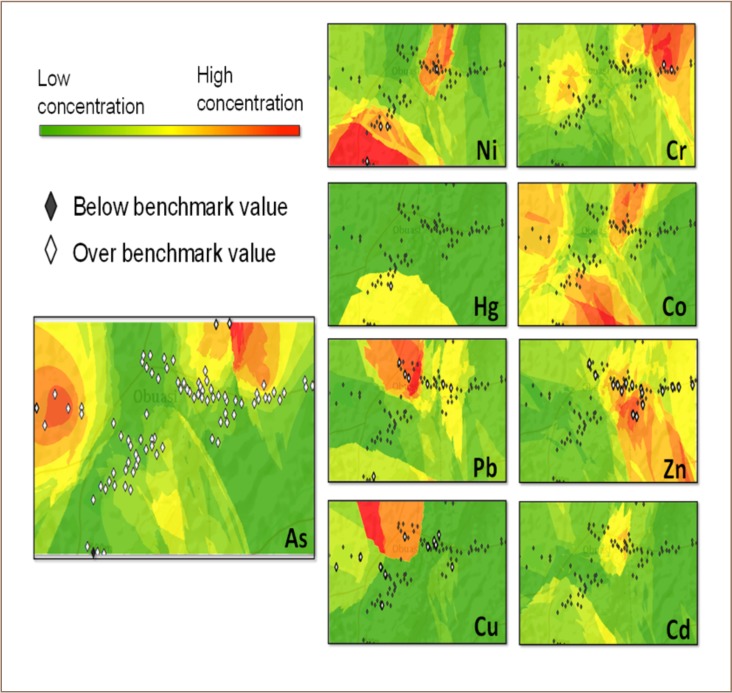
Heavy metals distribution map

The distribution map of As in topsoils within the Obuasi municipality showed that As is widespread throughout the communities, with the highest concentrations detected at the Pompura Treatment Plant tailings dam (*Figure 2*). Although levels exceeded recommended guidelines at all sites, the distribution of As showed two hotspots at the Sulfite Treatment Plant (Sulfite Treatment Plant) and Pompura Treatment Plant tailings dam (*Figure 2*). Nickel levels within the study area were generally low compared to recommended levels, but showed wide variation. Nickel recorded a hotspot around the tailings dams (Sulfite Treatment Plant and Pompura Treatment Plant) and Sanso (*Figures 1 and 2*). While Hg showed hotspots at Sanso, Cd did not show any appreciable variation in its distribution within the study area. As shown in Figure 2, Cu showed hotspots at Bediaso.

### Relationship between heavy metals and soil properties

Soil properties, such as pH and soil organic matter, play an important role in the mobility, adsorption, desorption, movement and bioavailability of heavy metals, thus influencing their distribution and biological availability in soils.^42–44^ This role is generally illustrated by good correlations between heavy metal concentrations and soil properties.^45^,^46^ Soil pH in the samples ranged from 6.35 (Brono Estate) to 7.31 (Brahabebome), with a mean value of 6.73 ± 0.48, whereas the soil organic matter content ranged from 3.43 (Kwabrafosu) to 10.3% (Bill Huston) (*Supplemental Material 1*).

### Sources of metals in soil

**Figure 3:**
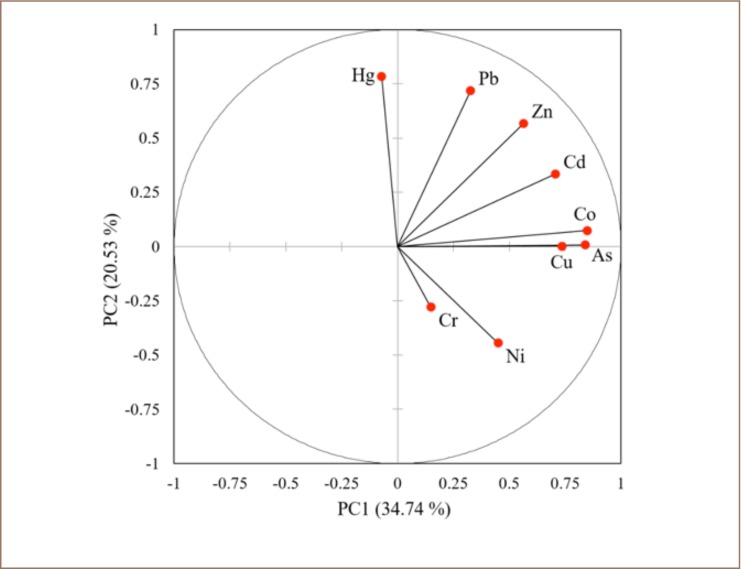
Distribution of heavy metals and a metalloid in soils in Obuasi, Ghana

Three principal components (PC1, PC2, and PC3) were extracted, accounting for 66.9% of the total variance. As shown in Figure 3, PC1, the most important component, explained 35% of the total variance and was characterized by high loadings of As, Co, Cu, Zn, Cd, and Pb.

Nickel and Cr were grouped under PC2 and made up 21% of the component. PC3 explained 11.6% of the total variance and was dominated by high loading of Hg.

### Soil contamination risks

Table 2 shows the C^i^_f_ and C_deg_ of metals and their contributions to the total potential ecological risk of surface soils. The average C^i^_f_ of Cu, Ni and As indicated high pollution (C^i^_f_ > 3), while Hg indicated moderate pollution. The C_deg_ of metals ranged from 25.4 (Boete) - 130 (Pompura Treatment Plant), with an average of 61.9 ± 28.2. As shown in Table 2, all of the communities including the two tailing dams were very highly polluted with metals (*Tables 1 and 2*).

**Table 2:** Metal Pollution Coefficient and Integrated Pollution Level of Heavy Metals and a Metalloid in Surface Soils at Obuasi, Ghana

Sample site	Hg	Cu	Zn	Pb	Cd	Ni	As	Cr	Cdeg
PTP tailings dam	0.38	**6.67**	0.88	0.31	0.26	**16.2**	**103**	**2.61**	**130**
STP tailings dam	0.21	**1.48**	0.40	0.22	0.30	**17.1**	**54.6**	**1.02**	**75.4**
Sanso	**21.6**	**1.38**	0.87	**1.68**	0.13	**17.6**	**15.4**	0.63	**59.3**
Sam Jona Estate	0.33	**4.76**	0.73	0.35	0.10	**1.13**	**52.2**	**3.10**	**62.7**
Bruno Estate	0.29	**1.25**	0.50	0.36	0.18	**3.22**	**70.5**	**1.97**	**78.2**
Danquah	0.21	**1.09**	0.48	0.08	0.10	**9.43**	**43.9**	**1.32**	**56.6**
Anyinam	**1.29**	**1.55**	0.83	0.59	0.25	**10.7**	**82.7**	**1.55**	**99.5**
Bill Huston	0.46	0.90	0.51	0.29	0.07	**5.79**	**26.7**	0.78	**35.5**
Pomposo	0.46	0.90	**1.28**	**1.05**	0.05	**3.88**	**20.0**	**5.09**	**32.7**
Akaporiso	0.71	**1.26**	**1.83**	0.53	0.13	**3.75**	**25.3**	**1.25**	**34.8**
Bossman	0.54	**1.23**	0.55	0.13	0.08	**4.93**	**33.4**	**1.22**	**42.1**
Brahabebome	**1.57**	0.69	**1.34**	0.32	0.22	**3.07**	**29.1**	**2.36**	**38.7**
Boete	**2.44**	0.62	0.70	0.17	0.07	**2.46**	**17.7**	**1.13**	**25.4**
Ahansonyewudia	**5.09**	**2.10**	**2.31**	**1.42**	0.27	**6.12**	**31.0**	**1.84**	**50.1**
Esikafuoabantem	**1.32**	**11.2**	**2.38**	0.25	0.08	**11.5**	**63.4**	0.78	**91.0**
Tutuka	**2.38**	**2.26**	**1.68**	**2.27**	0.28	**12.9**	**66.0**	**1.23**	**89.1**
Central Market	0.75	**1.91**	**2.01**	**1.57**	0.64	**2.46**	**45.1**	0.61	**55.1**
Wawasi	**1.80**	**1.20**	**1.26**	0.33	0.28	**2.97**	**28.7**	**2.34**	**38.9**
Kwabrafosu	**3.33**	**1.46**	**1.72**	**1.28**	0.31	**1.44**	**69.1**	0.64	**79.3**
Estate	0.67	**2.80**	**1.24**	0.49	0.38	**2.25**	**23.6**	**2.32**	**33.7**
Bediaso	**4.08**	**59.6**	**1.01**	**3.63**	0.44	**2.93**	**32.2**	**1.54**	**105**
Bongobri	1.00	**1.20**	**1.54**	**1.19**	0.28	**2.55**	**38.4**	**1.86**	**48.1**

Average	2.32	4.89	1.18	0.84	0.22	6.57	44.2	1.69	61.9
SD	4.51	12.4	0.61	0.87	0.15	5.40	23.3	1.03	28.2
Minimum	0.21	0.62	0.40	0.08	0.05	1.13	15.4	0.61	25.4
Maximum	21.6	59.6	2.38	3.63	0.64	17.61	103	5.09	130

*Abbreviations: C^i^_f_*, metal pollution coefficient; C_deg_, integrated pollution level. Bold indicates high *C^i^_f_* and C_deg_ values (i.e. middle to high pollution) based on: (a) low (*C^i^_f_ ≤* 1), middle (1 < *C^i^_f_ ≤* 3) or high (*C^i^_f_* > 3).^33^ (b) C_deg_ < 5, low pollution; 5 ≤ C_deg_ < 10, medium pollution; 10 ≤ C_deg_ < 20, high pollution; and C_deg_ ≥ 20, very high pollution.^34^

### Human health risks

Arsenic was the most abundant metal in the present analysis. The overall average concentration of As in the study area (256 ± 135 mg/kg) was more than 14 times higher than the USEPA recommended value (18 mg/kg) (*Table 1*). Further, the calculated HQs for Cu, Zn, Pb, Ni and Co were less than 1 for both children and adults from all study sites (data not shown).

As shown in Table 3, the HQ for As ranged from 0.955 to 6.37 (children) and 0.102 to 0.683 (adults). Ingestion of soils in 95% of the communities in the study area could pose non-carcinogenic health risks to children. Some of the communities with the highest HQ were Anyinam (5.11), Bruno Estate (4.35), Kwabrafosu (4.27), and Tutuka (4.08), etc. The average HQ in the communities (without tailing dams) for both children and adults was 2.51 ± 1.23 and 0.270 ± 0.132, respectively.

**Table 3:** Human Health Risk Characterization for Arsenic in Obuasi Soils

	Hazard Quotient		Cancer risk	

Sample sites	Child (As)	Adult (As)	Child (As)	Adult (As)
PTP tailings dam	6.37	0.683	2.87E-03	3.08E-04
STP tailings dam	3.37	0.362	1.52E-03	1.63E-04
Sanso	0.955	0.102	4.30E-04	4.62E-05
Sam Jona Estate	3.22	0.346	1.45E-03	1.56E-04
Bruno Estate	4.35	0.467	1.96E-03	2.10E-04
Danquah	2.71	0.291	1.22E-03	1.31E-04
Anyinam	5.11	0.548	2.30E-03	2.47E-04
Bill Huston	1.65	0.177	7.43E-04	7.98E-05
Pomposo	1.23	0.132	5.56E-04	5.97E-05
Akaporiso	1.56	0.168	7.05E-04	7.56E-05
Bossman	2.06	0.221	9.30E-04	9.98E-05
Brahabebome	1.80	0.193	8.10E-04	8.70E-05
Boete	1.09	0.117	4.94E-04	5.30E-05
Ahansonyewudia	1.91	0.205	8.63E-04	9.26E-05
Esikafuoabantem	3.92	0.420	1.76E-03	1.89E-04
Tutuka	4.08	0.437	1.84E-03	1.97E-04
Central Market	2.79	0.299	1.26E-03	1.35E-04
Wawasi	1.77	0.190	8.01E-04	8.59E-05
Kwabrafosu	4.27	0.458	1.92E-03	2.06E-04
Estate	1.45	0.156	6.57E-04	7.05E-05
Bediaso	1.99	0.213	8.97E-04	9.62E-05
Bongobri	2.37	0.255	1.07E-03	1.15E-04

Average	2.73	0.293	1.23E-03	1.32E-04
SD	1.44	0.154	6.48E-04	6.96E-05
Median	2.22	0.238	1.00E-03	1.07E-04
Minimum	0.955	0.102	4.30E-04	4.62E-05
Maximum	6.37	0.683	2.87E-03	3.08E-04

*Abbreviations:*SD, standard deviation

The cancer risk posed by As for children ranged from 4.30 × 10^−4^ to 2.87× 10^−3^ (*Table 3*). Based on the USEPA recommended range of 10^−6^ to 10^−4^, the cancer risk for children (greater than 10^−3^) was higher in 45% of the communities. Anyinam (2.3 × 10^−3^) recorded the highest cancer risk, followed by Bruno Estate (1.96 × 10^−3^), Kwabrafosu (1.92 × 10^−3^), Tutuka (1.84 × 10^−3^), Esikafuoanbantem (1.76 × 10^−3^), Sam Jona Estate (1.45 × 10^−3^), Central market (1.26 × 10^−3^), Danquah (1.22 × 10^−3^) and Bongobri (1.07 × 10^−3^). The average cancer risk assessment based on soil ingestion in the studied communities was higher for children (1.13 × 10^−3^) than adults (1.22 × 10^−4^). The carcinogenic risk of As for adults through ingestion of soil in the studied communities ranged from 4.62 × 10^−5^ to 2.47 × 10^−4^ at Sanso and Anyinam, respectively (*Table 3*).

## Discussion

The high levels of As in the study area could be due to the nature of the gold bearing ore which is comprised of mineralized pyrites and arsenopyrites.^47^ Arsenic is a toxic substance and due to its non-biodegradable nature, it can accumulate in surface soils and lead to contamination of food crops, surface and ground water. It can also enter the food chain through plant assimilation. The concentrations of As in communities in the present study far exceeded those recorded in a previous study by Amonoo-Neizer et al. at Kwabrafoso (48.9 ± 10.9 mg/kg dw).^47^

Lead concentrations in soils were high in some communities, but compared to average concentrations in urban soils worldwide, the mean value (43.8 ± 45.2 mg/kg dw) in the present study was low compared to values reported in large and industrialized cities such as Palermo (Sicily) (202 mg/kg) and Rome (330 mg/kg).^48^,^49^ However, compared to unpolluted soils, Pb levels were higher in 23% of the communities (*Supplemental Material 1*).^30^ The presence of elevated levels of Pb in soil from some communities, including the central market, is not surprising because Pb has greater soil retention and adsorption capacity than any other metal. Lead is therefore considered immobile in sub-surface soil and generally gets accumulated in surface soils when deposited.^50^

The concentrations of soil Hg determined in some areas (Sanso and Boete) in this study were higher than those reported in previous studies, and may be due to recent Hg contamination.^47^ The concentrations at some sites coupled with wide coefficients of variation (coefficients of variation of 194%; *Supplemental Material 1*) suggest anthropogenic sources, since Hg is used to amalgamate gold from ore.

### Distribution maps

The high concentrations or hotspots at some sites could be attributed to artisanal and small-scale gold mining activities. Illegal mining activities are frequently practiced in some communities and this could be the source of soil heavy metals pollution. The distribution pattern of Pb and Cu showed hotspots around the city center (central market), with higher levels than recommended for Pb.

### Heavy metals and soil properties

Metal mobility and availability are generally higher in acidic soil conditions. The thresholds for the solubility and uptake of metals such as Zn, Ni As and Cr occur between pH of 4.5 and 6.42. The pH of the soil within the study area (6.35 to 7.31) was above the threshold for heavy metal mobility and therefore would have no significant effect on the mobility and availability of heavy metals in soil. When metals are bound to organic matter, the toxic effects may be pronounced, because the metal becomes more bioavailable.^51^ No metals in the study area showed any significant correlation with pH and soil organic matter (*Table 4*), similar to the results by Manta et al. and Al–Khashman and Shawabkeh.^48^,^52^ This could be attributed to the narrow range of pH (near neutrality) in the samples. Additionally, lack of significant correlation between soil properties and heavy metals could be attributed to a continuous input since the release and transport of heavy metals are complex processes.^46^,^53^,^54^ Another possible explanation could be variations in soil type within the sampling area.^46^,^54^

**Table 4:** Correlation Between Metal Concentrations, Soil Organic Matter and Soil pH

	Hg	Cu	Zn	Pb	Cd	Co	Ni	As	Cr	SOM	pH
Hg	1.00										
Cu	−0.05	1.00									
Zn	−0.29	0.01	1.00								
Pb	−0.12	0.68^*^	0.32	1.00							
Cd	0.11	0.30	0.32	0.52^*^	1.00						
Co	−0.05	−0.10	−0.13	0.03	0.17	1.00					
Ni	0.48	−0.09	−0.17	−0.02	−0.13	0.73^*^	1.00				
As	0.07	−0.02	−0.02	−0.10	0.17	0.66^*^	0.39	1.00			
Cr	−0.17	−0.03	−0.08	−0.10	−0.19	−0.20	−0.25	−0.07	1.00		
SOM	−0.10	−0.16	−0.13	0.06	−0.30	−0.43	−0.20	−0.39	−0.11	1.00	
pH	−0.14	−0.11	0.11	0.12	−0.16	−0.15	0.03	−0.17	0.35	0.01	1.00

^*^correlation is significant at the *p* < 0.05 level

### Source identification

Lead was highly distributed around the city center where vehicular traffic is very dense and therefore lead pollution could be related to vehicular traffic. The use of leaded fuel in the past may account for the high levels of Pb in soil in the central part of the city. Even though the use of leaded petrol has ceased in Ghana, Pb is highly immobile and could remain strongly bound to soil. Pollution with the other metals, Cu and Zn, were more likely to have come from the wear and tear of tires, engine and brakes, and therefore their concentrations are expected to increase in this area since vehicular traffic is heavy. Additionally, the presence of Zn in the environment is associated with mining and smelting, which pollutes the air, water and soil, which ultimately undergoes oxidation to release Zn^2+^ ions.^55^

Levels of As in soils could have resulted from blasting of gold bearing rock, which is the most common method of obtaining ore. Miners engage in surface and sub-surface mining.^56^ Arsenic levels in soils could also be due to the nature of the gold bearing ore, which is comprised of mineralized pyrites and arsenopyrites. Processing of the ore involves roasting, and this results in the production of arsenic trioxide gas which is distributed throughout the study area by air currents.

The possible sources of Cr in soils from Obuasi could be due to weathering of the rock system. Other sources of Cr in the study area could be the occasional discharge of acid industrial wastes or mine drainage.^55^

In Ghana, amalgamation using Hg is the preferred gold recovery method employed by almost all artisanal gold miners because it is a very simple and inexpensive technique.^47^ The high levels of Hg in soils could therefore be due to contamination from mining processes.

### Ecological risk assessment

The very high pollution (C_deg_ ≥ 20) found in the present study could be due to the proximity of some sample sites to the mines, as we observed very high C_deg_ values from the two tailings dams. In addition, high pollution levels could be attributed to the several artisanal and small-scale mining activities within some communities.

The E^i^_r_ of As showed that 59% of sampled sites posed potentially high risks to the surrounding ecological system. Mercury was found to pose moderate to very high risks in 45% of the sampled sites (*Tables 1 and 5*). As shown in Table 5, the RI at all study sites ranged from 260 (Pomposi) - 1176 (Pompura Treatment Plant), with an average of 607 ± 276. According to the classification by Hakanson (*Table 1*), soils in 41% of all study sites pose very high risks to the ecological system, 50% pose considerable risk, and 9% pose moderate risks to the ecological system.^31^ Arsenic and Hg on average contributed 73 and 15%, respectively (total contribution 88%), of the RI. This trend is similar to that in a study by Bortey-Sam et al., in which As and Hg contributed 85% of the total RI in agricultural soils in Tarkwa, Ghana.^12^ However, in that study, the contribution from As was 10%, and 75% for Hg.^12^ The average RI of heavy metals and a metalloid from all study sites in Obuasi (607 ± 276) indicated very high risk (*Tables 1 and 5*) to the ecological system, including mammals, higher than that reported by Bortey-Sam et al. in Tarkwa soils.^12^

**Table 5:** Monomial Potential Ecological Risk Factor and Potential Ecological Risk of Heavy Metals and a Metalloid in Surface Soils in Obuasi, Ghana

Sample site	Hg	Cu	Zn	Pb	Cd	Ni	As	Cr	RI
PTP tailings dam	15.0	33.3	0.88	1.57	7.69	**81.3**	**1031**	5.21	**1176**
STP tailings dam	8.4	7.40	0.40	1.10	8.92	**85.8**	**546**	2.03	**660**
Sanso	**865**	6.90	0.87	8.42	3.85	**88.0**	**154**	1.25	**1129**
Sam Jona Estate	13.3	23.8	0.73	1.74	3.08	5.66	**522**	6.20	**576**
Bruno Estate	11.6	6.24	0.50	1.81	5.38	16.1	**705**	3.94	**750**
Danquah	8.33	5.43	0.48	0.41	3.08	**47.1**	**439**	2.63	**507**
Anyinam	**51.6**	7.74	0.83	2.97	7.62	**53.5**	**827**	3.09	**955**
Bill Huston	18.3	4.52	0.51	1.46	2.00	28.9	**267**	1.55	**324**
Pomposo	18.3	4.48	1.28	5.26	1.54	19.4	**200**	10.2	**260**
Akaporiso	28.3	6.31	1.83	2.67	3.85	18.7	**253**	2.50	**317**
Bossman	21.6	6.14	0.55	0.67	2.31	24.6	**334**	2.43	**392**
Brahabebome	**62.8**	3.45	1.34	1.61	6.54	15.3	**291**	4.72	**387**
Boete	**97.7**	3.10	0.70	0.85	2.00	12.3	**177**	2.27	**296**
Ahansonyewudia	**203**	10.4	2.31	7.11	8.08	30.6	**310**	3.67	**576**
Esikafuoabantem	**52.6**	**56.1**	2.38	1.27	2.31	**57.6**	**634**	1.57	**808**
Tutuka	**95.1**	11.3	1.68	11.3	8.46	**64.6**	**660**	2.45	**855**
Central Market	30.0	9.57	2.01	7.85	19.2	12.3	**451**	1.23	**533**
Wawasi	**72.1**	6.02	1.26	1.63	8.46	14.8	**287**	4.68	**397**
Kwabrafosu	**133**	7.29	1.72	6.38	9.23	7.22	**691**	1.27	**857**
Estate	26.6	14.0	1.24	2.44	11.5	11.2	**236**	4.64	**308**
Bediaso	**163**	**298**	1.01	18.1	13.1	14.6	**322**	3.08	**834**
Bongobri	40.0	6.02	1.54	5.94	8.46	12.7	**384**	3.72	**462**

Average	92.6	24.4	1.18	4.21	6.67	32.8	442	3.38	607
SD	180	62.3	0.61	4.35	4.36	26.9	233	2.07	276
Minimum	8.33	3.10	0.40	0.41	1.54	5.66	154	1.23	260
Maximum	865	298	2.38	18.1	19.2	88.1	1031	10.2	1176

Bold indicates *E^i^_r_* (monomial potential ecological risk factor) and RI (potential ecological risk) indicates moderate to high risk of heavy metals and/or metalloid.

### Human health risks

The human health risk assessment for children determined HQs suggesting potential non-carcinogenic risks due to As exposure (*Table 3*). As shown in Table 3, HQs for children throughout the study area were high (average of 2.73 ± 1.44; range 0.955–6.37). In the communities without tailing dams, the average HQ ranged from 0.955 (Sanso) to 5.11 (Anyinam), with an average of 2.51 ± 1.23. Remedial action and control methods are therefore required in the communities to further avoid the deleterious effects of As exposure. However, the HQs for adults were all below 1, mainly due to behavioral differences. The high HQs detected for children could be due to their frequent hand-to-mouth or object-to-mouth activities, which could increase toxicant exposure, including As.^57^,^58^ Previous studies have found that children typically ingest an average of 50 mg/d of soil.^59^ However, in the case of pica, this amount could be higher.^60^,^61^

The present study further revealed that the cancer risk due to As exposure was higher for children (1.13 × 10^−3^) compared to adults (1.22 × 10^−4^). Based on these results, there could be potential cancer risks for Obuasi residents, especially children. The high non-cancer and cancer risks detected in the communities, particularly Anyinam, Bruno Estate, Sam Jona Estate and Danquah could be due to their proximity to the Sulfite Treatment Plant tailings dam (*Figure 1*), in addition to other artisanal and small-scale mining activities within the study areas. In Kumasi, Ghana, Darko et al. reported that the cancer risk (based on maximum exposure point concentration) for As due to soil ingestion was 3.22 × 10^−5^ for children and 1.73 × 10^−5^ for adults.^37^

## Conclusions

The investigation of soil samples in the Obuasi municipality has revealed high accumulations of As in surface soils and levels exceeded background concentrations throughout the study area. Distribution maps for As highlighted two hotspots around the tailing dams, while Pb, Cu and Zn were elevated in the central area of the Obuasi municipality. Principal component analysis indicated that mining activities have played a significant role in the levels of some metals in Obuasi soils. The RI of the results showed that the investigated soils could pose very high risks to the surrounding ecological system due to metal exposure, and As and Hg contributed 88% of these risks. The current study found that ingestion of As-contaminated soils could pose both non-cancer and cancer risks to Obuasi residents, especially children.
