# Supplementary material for: Characterization, Spatial Variation and Risk Assessment of Heavy Metals and a Metalloid in Surface Soils in Obuasi, Ghana
Source: J Health Pollut. 2018 Aug 20;8(19):180902. doi: 10.5696/2156-9614-8.19.180902 (PMC6257175; doi:10.5696/2156-9614-8.19.180902)
Supplement: Supplementary file 1 [file Akoto_supplemental_material.pdf]

Supplemental Material

Characteristics of Heavy Metals and a Metalloid in Soil in Obuasi, Ghana

n		Soil pH	WC%	SOM%	Hg	Cu	Zn	Pb	Cd	Co	Ni
15	Mean	6.79	4.59	3.92	0.09	**140**	63.1	16.3	0.1	**25.7**	109
	SD	0.538	5.63	1.35	0.1	235	24.6	18.7	0.07	10.1	166
10	Mean	6.55	5.97	5.19	0.05	31.1	28.7	11.4	0.116	14	115
	SD	0.214	5.57	2.66	0.03	18.9	22.1	10	0.243	15.4	140
4	mean	6.91	11.4	6.52	5.19	29	62.8	**87.6**	0.05	**23.9**	118
	SD	0.823	4.52	0.899	7.28	5.88	27.6	123	0.02	20.9	192
3	mean	6.59	12.7	7.69	0.08	**100**	52.3	18.1	0.04	6.85	7.58
	SD	0.14	10.2	5.88	0.05	116	17.5	4.99	0.01	1.25	2.41
3	mean	6.35	5.40	9.06	0.07	26.2	35.7	18.8	0.07	13.5	21.6
	SD	0.601	4.50	1.73	0.01	6.25	15.7	5.96	0.02	10.4	11.7
3	Mean	6.52	5.06	8.05	0.05	22.8	34.5	4.3	0.04	8.02	63.2
	SD	0.321	2.10	4.64	0.01	9.46	23.7	0.719	0.04	9.25	16.6
3	Mean	6.8	2.57	4.84	0.31	32.5	59.6	30.9	0.099	**20.3**	71.8
	SD	0.645	2.07	0.911	0.29	15.7	36.9	18.9	0.063	16.9	47.9
3	mean	6.64	12.1	10.25	0.11	19	37	15.2	0.026	3.13	38.8
	SD	0.055	8.22	0.962	0.03	3.56	12.2	8.04	0.014	0.761	15.8
3	mean	7.29	8.97	6.88	0.11	18.8	91.8	54.7	0.02	1.21	26
	SD	0.374	4.19	2.84	0.05	5.48	62.1	35.7	0.01	0.49	11
3	mean	6.91	3.95	8.58	0.17	26.5	**132**	27.8	0.05	1.42	25.1
	SD	0.087	1.52	3.88	0.07	8.22	64.4	17.4	0.02	0.263	2.3
3	mean	7.03	6.65	6.94	0.13	25.8	39.3	6.94	0.03	2.68	33
	SD	0.534	0.489	2.76	0.06	8.4	12.8	4	0.04	1.59	20.9
3	mean	7.31	6.13	6.46	0.377	14.5	96.8	16.7	0.085	0.806	20.6
	SD	0.28	5.99	4.46	0.364	3.89	64.7	18.7	0.049	0.276	12.4
3	mean	6.49	4.98	6.08	0.586	13	50.6	8.79	0.026	0.839	16.5
	SD	0.530	3.53	2.13	0.731	1.87	32.5	9.33	0.018	0.314	10
2	mean	6.73	3.70	6.93	1.221	44	**166**	**73.9**	0.105	3.43	41
	SD	0.014	3.02	1.54	0.326	18.5	1.25	26.4	0.07	1.66	3.32
3	mean	6.34	1.94	4.18	0.316	**236**	**171**	13.2	0.03	11.7	77.3
	SD	0.320	1.02	0.72	0.304	25	186	9.58	0.01	10.2	85.5
3	mean	6.99	4.18	9.56	0.571	47.5	**121**	**118**	0.11	13.3	86.6
	SD	0.804	3.07	10.8	0.336	12	3.79	108	0.076	9.12	56.8
4	mean	6.53	4.10	6.74	0.18	40.2	**145**	**81.6**	0.25	10.6	16.5
	SD	0.259	3.23	4.06	0.113	30.2	106	72.7	0.353	13.6	12
3	mean	6.85	2.38	3.99	0.433	25.3	90.6	17	0.11	9.95	19.9
	SD	0.766	1.41	1.96	0.482	9.8	50.3	5.98	0.106	6.97	13.1
3	mean	6.96	1.31	3.43	0.8	30.6	**124**	66.4	0.12	9.69	9.68
	SD	0.571	0.0975	1.28	0.604	13.6	18.8	75.2	0.089	5.89	7.65
3	mean	6.51	2.08	4.18	0.16	58.83	89	25.4	0.15	5.65	15.1
	SD	0.263	0.544	0.302	0.126	49.6	29	5.2	0.086	1.34	5.21
3	mean	6.66	2.64	5.44	0.98	**1253**	72.8	**189**	0.17	3.59	19.6
	SD	0.395	1.45	2.12	1.23	1755	46.1	131	0.105	2.24	10.1
